# Systemic postnatal corticosteroid use for the prevention of bronchopulmonary dysplasia and its relationship to early neurodevelopment in extremely preterm infants

**DOI:** 10.1007/s12519-023-00708-8

**Published:** 2023-03-27

**Authors:** Emilia Esterman, Traci-Anne Goyen, Pranav Jani, Gemma Lowe, Jane Baird, Rajesh Maheshwari, Daphne D’Cruz, Melissa Luig, Dharmesh Shah

**Affiliations:** 1 Department of Neonatology, Westmead Hospital, Westmead, Australia; 2 Faculty of Medicine and Health, The University of Sydney, Sydney, Australia

**Keywords:** Corticosteroids, Early neurodevelopment, General movement assessment, Preterm infants

## Abstract

**Background:**

Systemic postnatal corticosteroid use in extremely preterm infants poses a risk of adverse neurodevelopmental outcomes. This study explores their use beyond seven days of age with early neurodevelopmental assessments during the fidgety period (9–20 weeks postterm age).

**Methods:**

This retrospective single-center cohort study included inborn extremely preterm infants from 1 January 2014 to 31 December 2018. Outborn infants, those with congenital or genetic abnormalities, and those who received postnatal corticosteroids for nonrespiratory reasons were excluded. The cohort was dichotomized based on the status of corticosteroid receipt. Early neurodevelopmental outcomes were reported using Prechtl’s General Movements Assessment.

**Results:**

Of the 282 infants, 67 (23.75%) received corticosteroids. Of these, 34 (50.75%) received them for dependency on invasive ventilation (intermittent positive-pressure ventilation), and the remainder received them for dependency on non-invasive ventilation continuous positive airway pressure (CPAP) or bi-level positive airway pressure (BiPAP). Abnormal or absent fidgety movements were observed in 13% of infants (7/54) who received corticosteroids compared to 2% of infants (3/146) who did not. An increased odds for an abnormal general movements assessment from corticosteroid use after adjusting for gestational age [adjusted odds ratio (aOR) = 5.5, 95% confidence interval (CI) = 1.14–26.56] was observed. The motor optimality scores differed between the two groups [corticosteroid group: 25.5 (23–26) versus no-corticosteroid group: 26 (24–28); *z* =  − 2.02]. A motor optimality score < 20 was observed in 14.8% of infants (8/54) in the corticosteroid group compared to 2% of infants (3/146) in the noncorticosteroid group. This difference was significant after adjustment for gestational age (aOR 5.96, 95% CI 1.28–27.74).

**Conclusions:**

Abnormal early neurodevelopment was observed in infants who received systemic postnatal corticosteroids. The relationship between these findings and other factors influencing early neurodevelopment needs further exploration.

## Introduction

Bronchopulmonary dysplasia (BPD) is the most prevalent morbidity in convalescing preterm infants [1–3]. In Australia and New Zealand, it affects approximately 50% of extremely preterm infants (born < 28 weeks gestational age) [1, 4–8]. For this study, the Australian and New Zealand Neonatal Network's definition for BPD was used. BPD was defined as the ongoing need for supplemental oxygen and/or respiratory support at or beyond 36 weeks postmenstrual age (PMA) [1]. Increased survival of extremely preterm infants and provision of active care to infants as young as 22 weeks at birth may have increased its prevalence [1, 7, 9, 10]. While BPD is complex and multifactorial in origin, inflammation of the lungs plays a key role in its pathogenesis [4, 7, 11–14]. Postnatal corticosteroids (PNC), including dexamethasone, are used in the management of BPD due to their anti-inflammatory properties. However, there is controversy regarding their impact on long-term neurodevelopment [3, 4, 7, 10, 12, 15–17].

While the American Academy of Pediatrics and other organizations recommend using a course of low-dose dexamethasone for ventilator-dependent preterm infants, there are no formal recommendations for its use in preterm infants with evolving BPD who are dependent on noninvasive respiratory support [5, 9, 10, 12, 18, 20]. Using PNC in the first seven days of life is associated with short- and long-term adverse effects, such as gastrointestinal perforation and cerebral palsy (CP) [11, 21, 22]. Prechtl’s General Movements Assessment (GMA), conducted during the fidgety period of development (9–20 weeks postterm age), is a simple method of assessing infant neurodevelopment. It is a sensitive early marker used to identify infants at risk of developing CP [23–29]. Reports exploring the association between PNC use and GMAs during the fidgety period of development are scarce.

Our unit’s current practice is to administer systemic PNC to a select group of extremely preterm infants after seven days of age either to facilitate extubation or prevent reintubation on the background of evolving BPD. Using a shorter course and a lower dose of PNC beyond seven days of life may reduce long-term adverse effects [4–8, 11, 12, 16, 30, 31]. In our unit, low-dose dexamethasone (a cumulative dose of 0.89 mg/kg over 10 days) is used as per the Dexamethasone: a Randomized Trial (DART) protocol [32, 33]. At our institute, betamethasone is used as an antenatal corticosteroid and is administered to all women at risk of preterm birth (up to 34 weeks of gestational age). For infants with respiratory distress syndrome, poractant alfa is used for exogenous surfactant replacement as an early rescue therapy. The aim of this study was to explore the relationship between systemic PNC use and early neurodevelopment using Prechtl’s GMA. Based on concerns raised in the literature regarding a potential link between using PNC and adverse neurodevelopmental outcomes, we hypothesized that the neurodevelopmental outcomes for preterm infants who did not receive PNC would be better than for those who had received PNC [3, 4, 7, 10, 12, 15–17]. This has implications for improving practice in the use of PNC.

## Methods

### Study design

A single-center retrospective cohort study was conducted at a tertiary neonatal intensive care unit (NICU). All inborn preterm infants born before 29 weeks gestation and admitted to the NICU between 1 January 2014 and 31 December 2018 were identified from a prospectively maintained and verified Neonatal Intensive Care Units’ (NICUS) database and were included in the study. Infants with congenital or genetic abnormalities, those who received systemic steroids for nonrespiratory indications such as hypotension, and outborn infants were excluded. The Human Research Ethics Committee granted ethical approval (2108–15 QA). Obtaining informed consent from all research individuals was not required because this study retrospectively reviewed medical records. This database is a prospective data collection system of preterm infants (< 32 weeks) admitted to all tertiary NICUs in New South Wales (NSW) and the Australian Capital Territory (ACT) that collates maternal, perinatal and neonatal clinical data. These data are collected and verified by designated audit officers following standardized definitions for clinical outcomes. Clinical data were extracted from this database.

We dichotomized the cohort based on whether they had received PNC or not. Other variables potentially influencing neurodevelopmental outcomes, such as gestational age, small for gestational age (birth weight < 10th centile), major intraventricular hemorrhage (Grade III and IV based on Papile’s classification), histopathology-proven chorioamnionitis, antenatal steroids and antepartum magnesium sulphate administration, were included in the regression analysis [34].

Our unit's standard practice is to perform the GMA during the fidgety period of infant development (9–20 weeks postterm equivalent age). The infant’s general movements were captured by a short (2–3 minutes) video recording in the high-risk follow-up clinic. All videos were rated according to Prechtl’s method by two to four raters who had advanced training in GMAs. The raters were blinded to the infants’ PNC status and perinatal course. In case of disagreement, the rating was determined by the New South Wales General Movements Rater Network [24]. General movements were rated as normal, abnormal (exaggerated), or absent/sporadic fidgety. In addition, the infant’s motor repertoire was scored in detail using the motor optimality score revised (MOS-R). The quality of movement (observed movement patterns), repertoire (age-adequate movement repertoire), posture (observed postural patterns) and movement character were scored using the MOS-R. A motor optimality score (MOS) was then generated, which had a maximum score of 28 (the best possible) and a minimum score of 5. MOS-R is reported to be a good predictor of adverse neurodevelopmental outcomes [27, 28]. A MOS of 20–28 was considered an optimal score, and scores below 20 were considered suboptimal [27]. For analysis, GMAs were dichotomized to either normal or abnormal. An abnormal GMA result included abnormal or absent fidgety movements or a MOS score < 20. MOS was further classified as optimal range (20–28), suboptimal (9–19) and indicative of severe impairment (≤ 8).

### Data analysis

Descriptive statistics were used to summarize the study cohort characteristics, using the mean (standard deviation) for symmetrically distributed continuous variables and the median (interquartile range) for asymmetrically distributed variables. Numbers (percentages) were used to describe categorical data. Data were analyzed using Stata 17 (StataCorp, College Station, TX, USA). Differences between the groups for continuous data were tested using Student’s *t* test for symmetrically distributed data and the Mann-Whitney *U* test for asymmetrically distributed data. A chi-square test was used to report between-group differences in categorical variables. Binary multivariable logistic models were then created using backward stepwise selection to investigate the association for univariable models with a *P* value of < 0.2. The results from these models are reported with odds ratios and 95% confidence intervals. A two-tailed *P* value < 0.05 was considered significant. No adjustments have been made for multiple comparisons.

## Results

### Group characteristics

Between 1 January 2014 and 31 December 2018, we identified 282 eligible infants. Of these, 67 (23.75%) received PNC. PNC was administered for dependency on invasive ventilation (intermittent positive-pressure ventilation) in 34 infants (50.75%). Among the 33 infants (49.25%) received them for dependency on non-invasive ventilation continuous positive airway pressure (CPAP) or bi-level positive airway pressure (BiPAP), of which two infants were concurrently being weaned off noninvasive ventilation. Of the 67 infants who received PNC, 37 infants had a single course over 10 days, 21 had two courses, six had three courses and three had four courses. The first course of PNC was administered at a median age of 22 days [interquantile range (IQR) 13–34]. For the first course of PNC, the median age for infants dependent on invasive respiratory support was 16 days (IQR 10–24), compared to 23 days (14–25) for infants dependent on BiPAP and 39 days (25–61) for infants dependent on CPAP.

Demographic and baseline clinical characteristics of the cohort are shown in Table 1. Infants in the PNC recipient group were more premature (by two weeks) and were lighter at birth. In addition, this group had more small-for-gestational-age infants, histopathology-proven chorioamnionitis, infants who were intubated and received surfactant, and BPD. They also required both invasive and noninvasive ventilation for longer periods of time.

**Table 1:** Mother-infant dyad demographics and baseline clinical characteristics based on PNC status

Variables	PNC received group (*n* = 67)	No-PNC group (*n* = 215)	*P*
GA, wk, median (IQR)	25 (24–26)	27 (26–28)	< 0.001^‡^
BW, g, mean (SD)	778.6 (165)	977.8 (218)	< 0.001^‡^
Male sex, *n* (%)	42 (62.7)	109 (50.7)	0.085
Multiple births, *n* (%)	12 (17.9)	54 (25.1)	0.224
Maternal age, y, mean (SD)	31.6 (5.4)	31.8 (5.8)	0.802
GDM, *n* (%)	6 (9.0)	33 (15.4)	0.186
HDP, *n* (%)	16 (23.9)	42 (19.5)	0.442
SGA (BW < 10th centile), *n* (%)	11 (16.4)	11 (5.1)	0.003^†^
ANS, *n* (%)			
Complete	27 (40.3)	77 (35.8)	0.849
Incomplete	21 (31.3)	72 (33.5)	
None	4 (6.0)	10 (4.7)	
MgSO_4_, *n* (%)	49 (73.1)	140 (65.1)	0.223
Spontaneous preterm labour, *n* (%)	27 (40.3)	91 (42.3)	0.769
Maternal ABx, *n* (%)	42 (62.7)	131 (61.0)	0.797
Histopathological chorioamnionitis, *n* (%)	42 (62.7)	100 (46.5)	0.021*
Caesarean births, *n* (%)	36 (53.73%)	126 (58.6%)	0.480
Intubated at delivery for continuation of mechanical ventilation, *n* (%)	55 (82.1)	138 (64.2)	0.006^†^
Apgar score at 5 min, median (IQR)	7 (6–8)	7 (6–8)	0.863
Invasive ventilation, d, median (IQR)	9.2 (4.95–19.5)	0.7 (0.3–3.5)	< 0.001^‡^
Non-invasive ventilation, d, median (IQR)^a^	70.4 (57.2–91.1)	42.8 (24.6–57)	< 0.0001^‡^
Received surfactant, *n* (%)	65 (97)	184 (85.6)	0.011*
Deaths prior to discharge, *n* (%)	4 (6.0)	16 (7.4)	0.682
BPD, *n* (%)	55 (82.1)	70 (32.6)	< 0.001^‡^
Major IVH (Grade III/IV)^b^, *n* (%)	3 (4.5)	12 (5.6)	0.725
PVL, *n* (%)	3 (4.5)	2 (0.9)	0.055
Major surgery during hospital stay, *n* (%)^c^	28 (41.8)	24 (11.2)	< 0.001^‡^

*PNC* postnatal corticosteroid*, GA* gestational age, *BW* birth weight, *g* grams*, IQR* interquartile range*, SD* standard deviation*, GDM* gestational diabetes, *HDP* hypertensive disease of pregnancy, *SGA* small for gestational age, *ANS* antenatal steroids, *MgSO*_*4*_ magnesium sulfate, *ABx* antibiotics, *BPD* bronchopulmonary dysplasia, *IVH* intraventricular hemorrhage, *PVL* periventricular leukomalacia. ^*^P ≥ 0.01 and < 0.05, ^†^*P* ≥ 0.001 and < 0.01, ^‡^*P* < 0.001

^a^Non-invasive ventilation days include infants discharged home on CPAP and oxygen

^b^Major IVH (Grade III or IV) as per Papile's classification [34]

^c^Major surgery during hospital stay such as ligation of patent ductus arteriosus, laparotomy for necrotizing enterocolitis

### General movements assessment at 9–20 weeks post-term equivalent age

**Fig. 1:**
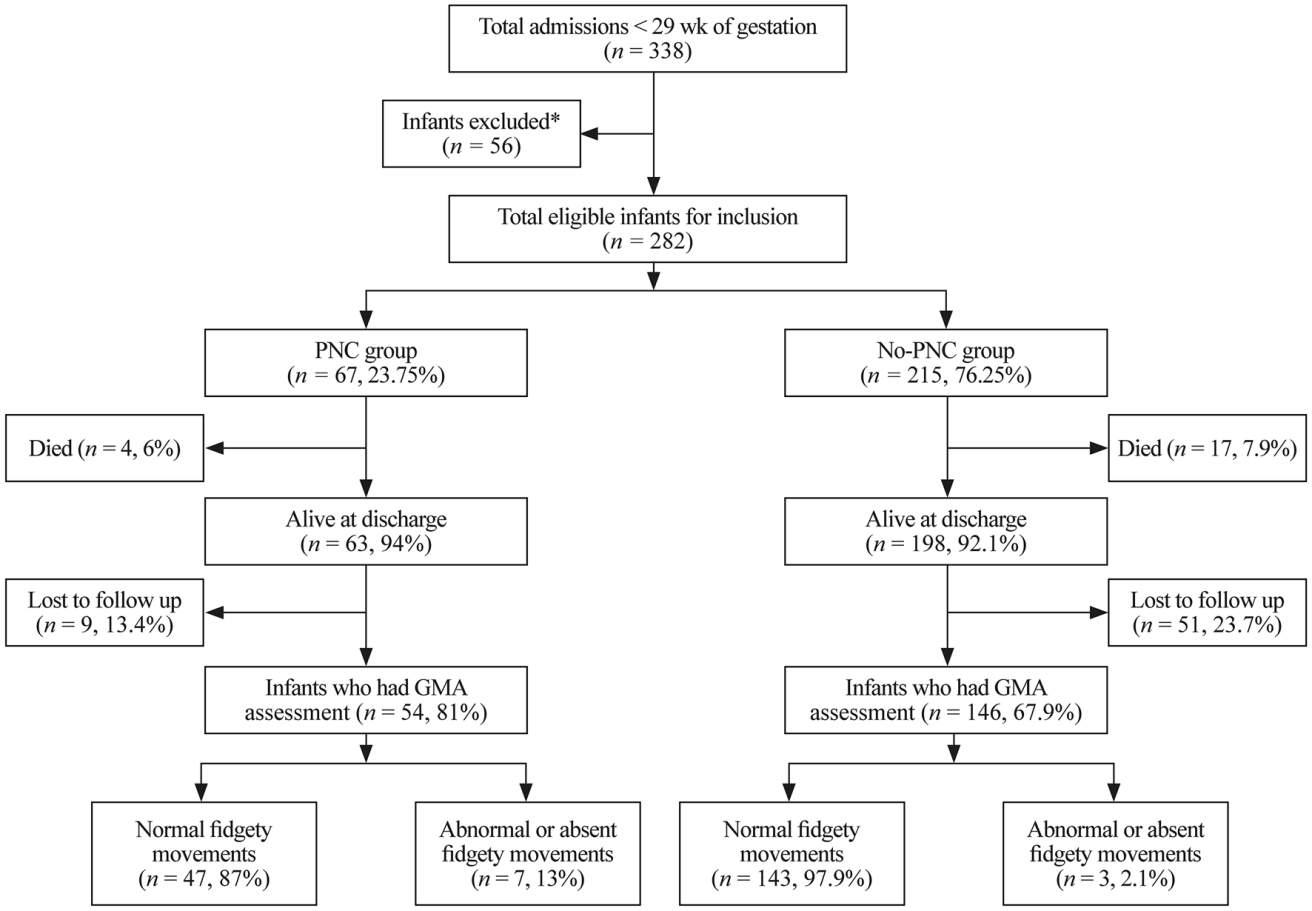
GMA outcomes and loss to follow-up based on corticosteroid status. *Infants met the study exclusion criteria. *PNC* postnatal corticosteroids, *GMA* General Movements Assessment

Of the 67 infants in the PNC recipient group, 54 (80.5%) infants had GMAs during the fidgety period. Of the 215 infants in the no-PNC group, 146 (67.9%) infants had GMAs during the fidgety period. Figure 1 provides details on GMA outcomes and loss to follow-up based on corticosteroid status.

Seven infants in the PNC recipient group (13%) had an abnormal GMA, while only three infants in the no-PNC group (2%) were abnormal [odds ratio (OR) 7.09, 95% confidence interval (CI) 1.76–28.56 and adjusted odds ratio (aOR) 5.5, 95% CI 1.14–26.56, *P* = 0.03] after adjusting for gestational age. The difference in the median MOS between the PNC recipient group and the no-PNC group was significant [25.5 (23–26) versus 26 (24–28); *P* = 0.043, *Z* =  −2.02]. Eight out of 54 infants (14.8%) in the PNC group had MOS < 20, while 3 out of 146 infants (2%) in the no-PNC group had MOS < 20; this difference was significant after adjustment for gestational age in logistic regression analysis (coefficient 1.78, 95% CI 0.24–3.32, *P* = 0.02). There were clear differences in MOS between the groups. In the PNC group, three (5.6%) infants fell into the range of severe impairment (MOS ≤ 8), and five infants (9.3%) fell into the suboptimal range (MOS 9–19), as opposed to no infants and three (2.1%) infants, respectively, in the no-PNC group. This difference was significant after adjustment for gestational age (aOR 5.96, 95% CI 1.28–27.74, *P* = 0.02).

Movements were further characterized and scored as summarized in Table 2. For quality of movement, there were four (7.4%) infants with more atypical movements (A) than normal movements (N) in the PNC group and four (2.7%) in the no-PNC group. The expected repertoire of movements was absent for 19 (35.2%) infants in the PNC group, as opposed to 33 (22.6%) in the no-PNC group. There was a higher proportion of infants in the PNC group with *N* < A for posture [6 (11.1%) versus 6 (4.1%)]. The only infant with cramped-synchronized movements was in the PNC group.

**Table 2:** Motor optimality score (MOS) components from General Movements Assessment (GMA) based on PNC status

Variables	PNC received group (*n* = 54)	No-PNC group (*n* = 146)
Abnormal or absent fidgety movements	7 (13%)	3 (2%)
MOS < 20	8 (14.8%)	3 (2%)
Quality of movement		
* N* > A	48 (88.9%)	136 (93.2%)
* N* = A	2 (3.7%)	6 (4.1%)
* N* < A	4 (7.4%)	4 (2.7%)
Movement repertoire		
Adequate	19 (35.2%)	52 (35.6%)
Reduced	16 (29.6%)	61 (41.8%)
Absent	19 (35.2%)	33 (22.6%)
Posture		
* N* > A	43 (76.9%)	131 (89.7%)
* N* = A	5 (9.3%)	9 (6.2%)
* N* < A	6 (11.1%)	6 (4.1%)
Movement characters		
Normal	25 (46.3%)	95 (65.1%)
Abnormal but not cramped-synchronized	28 (51.9%)	51 (34.9%)
Cramped-synchronized	1 (1.9%)	0

*PNC* postnatal corticosteroid*, N* normal, *A* atypical

For infants in the PNC recipient group, GMA outcomes were similar for single versus multiple courses of PNC. The median MOS for the group receiving multiple courses was 25.5 (IQR 23–26) versus 25 (IQR 21–26) for the single course group. There were no differences in the fidgety movements, repertoire or posture between the two groups. There were more movements of atypical quality in infants who received multiple courses of PNC [3 (12%) versus 1 (3%)]. One infant in the multiple course group and none from the single course group had cramped-synchronized movements.

We explored the effects of PNC use on GMAs for infants dependent on invasive versus noninvasive respiratory support within the PNC recipient group. Overall, PNC had similar effects on GMA, irrespective of the type of respiratory support. The median MOS for the group with invasive ventilation was 24 (IQR 21–26) versus 26 (IQR 23–28) for the noninvasive group; the difference was not significant. There were some differences in the quality of movements, with more atypical movements observed in the invasive ventilation group for fidgety movements [four (16%) versus three (10%)], quality of movement [three (12%) versus one (3%)], repertoire [10 (40%) versus 9 (31%)] and posture [four (14%) versus two (8%)]. The one infant with cramped-synchronized movement belonged to the invasive ventilation group.

### Relationship between postnatal corticosteroids status, clinical characteristics, and fidgety movement outcomes

The relationship between clinical characteristics and fidgety movement outcomes at GMA is shown in Table 3. On univariable analysis, the perinatal factors associated with increased odds for abnormal or absent fidgety movements were PNC administration (OR 7.1, 95% CI 1.77–28.56, *P* = 0.006), gestational age (OR 0.63, 95% CI 0.40–0.99, *P* = 0.047), birth weight (OR 0.99, 95% CI 0.99–0.99, *P* = 0.004), BPD (OR 10.88, 95% CI 1.35–87.61, *P* = 0.025), major intraventricular hemorrhage (OR 9.25, 95% CI 1.55–55.18, *P* = 0.015), periventricular leucomalacia (OR 15.58, 95% CI 2.28–106.72, *P* = 0.005) and major surgery (OR 4.28, 95% CI 1.18–15.57, *P* = 0.027). Of the infants with normal fidgety movements in the PNC group, 26 (55.32%) received one course of dexamethasone, 16 (34.04%) received two courses, three (6.38%) received three courses and two (4.26%) received four courses. For infants with abnormal or absent fidgety movements, four (57.14%) received one course of dexamethasone, and three (42.86%) received two courses. No infants in this group received three or four courses of dexamethasone. The multivariable logistic regression model is shown in Table 3. Although the adjusted odds ratio of abnormal or absent fidgety movements was higher in the PNC recipient group, this was not statistically significant. The presence of PVL significantly increased the odds of abnormal or absent fidgety movements.

**Table 3:** Relationship between perinatal variables and outcomes of fidgety movements from binary univariable and multivariable logistic regression analysis

Clinical characteristics	Univariable OR (95% CI)	*P* value	Multivariable aOR (95% CI)	*P* value
Received PNC	7.1 (1.76–28.56)	0.006^†^	3.980 (0.77–20.44)	0.098
GA (median, wk)	0.63 (0.40–0.99)	0.047*	NA	NA
BW (median, g)	0.99 (0.99–0.99)	0.004^†^	0.996 (0.991–1.000)	0.052
SGA	3.14 (0.60–16.23)	0.172	NA	NA
Female gender	1.02 (0.28–3.64)	0.974	NA	NA
Multiples	2.21 (0.59–8.19)	0.235	NA	NA
Chorioamnionitis	0.532 (0.41–5.60)	0.519	NA	NA
Received antenatal steroids (complete)	1.329 (0.21–8.21)	0.760	NA	NA
MgSO_4_	1.213 (0.30–4.84)	0.784	NA	NA
Spontaneous labour onset	1.235 (0.34–4.40)	0.745	NA	NA
Antibiotics in labour	1.00 (0.27–3.66)	1	NA	NA
Apgar score at 5 min (median)	0.800 (0.57–1.12)	0.193	NA	NA
Apgar score < 6 at 5 min	1.000 (0.12–8.32)	1	NA	NA
Intubated at birth for continuation of mechanical ventilation	4.571 (0.56–36.87)	0.154	NA	NA
ECM at birth	2.905 (0.32–26.20)	0.342	NA	NA
BPD	10.884 (1.35–87.61)	0.025*	NA	NA
Major IVH (Grade III/IV)	9.250 (1.55–55.18)	0.015*	5.758 (0.693–47.868)	0.105
PVL	15.583 (2.27–106.72)	0.005^†^	12.020 (1.111–130.086)	0.041*
Died before discharge	10.444 (0.86–126.21)	0.065	NA	NA
Major surgery during hospital stay	4.278 (1.17–15.565)	0.027*	NA	NA

*PNC* postnatal corticosteroids, *OR* unadjusted odds ratio from univariable model, *aOR* adjusted odds ratio from multivariable model, *GA* gestational age, *BW* birth weight, *g* grams, *SGA* small for gestational age, *MgSO*_*4*_ magnesium sulphate, *ECM* external cardiac massage for cardiopulmonary resuscitation, *min* minutes, *BPD* bronchopulmonary dysplasia, *IVH* intraventricular hemorrhage, *PVL* periventricular leukomalacia, *NA* variables excluded from multivariable regression analysis as *P* ≥ 0.2.

^*^P ≥ 0.01 and < 0.05

^†^*P* ≥ 0.001 and < 0.01

## Discussion

We explored the relationship between systemic PNC use and early neurodevelopmental assessment at 9–20 weeks postterm equivalent age in extremely preterm infants born < 29 weeks gestation. We observed that using PNC was associated with higher adjusted odds (adjusted for gestational age at birth) for an abnormal GMA in a univariable logistic regression but not in a multivariable regression model. Overall, GMA outcomes were similar for single versus multiple courses of systemic PNC, as well as for infants dependent on invasive versus noninvasive ventilation. A modest increase in the frequency of abnormal movements was observed for infants dependent on invasive ventilation.

Overall, about one-third of infants < 29 weeks gestation received systemic PNC for dependency on respiratory support. While the proportion of infants who received PNC for dependency on invasive ventilation was similar to regional and international practices [1, 22], there was considerable use of systemic PNC for dependency on noninvasive respiratory support. This practice might reflect the lack of evidence for PNC use in infants dependent on noninvasive ventilation. Doyle et al. described the relationship between the risk of BPD and death or CP [35]. Systemic PNC increased the risk of death or CP when the risk of BPD was below 35% and decreased its risk when the risk of BPD exceeded 65% [35]. The meta-regression analysis in Doyle’s study suggests reserving the use of systemic PNC for ventilator-dependent infants with a predicted risk of BPD ≥ 50%, which is supported in the recent literature [2, 6, 16, 22].

Our results support findings in previous studies in relation to risk factors for BPD [2, 3, 6, 7, 11, 13, 30, 36]. Interestingly, although perhaps reflective of the small sample size and dissimilar to previous studies, male sex was not found to be a significant risk factor for BPD [2, 13, 14, 37]. Gestational age has the strongest association with BPD, so it could be extrapolated that the lower the gestational age, the more likely it is that PNC is prescribed [2, 13].

The strongest predictors of a later diagnosis of CP on the MOS are absent fidgety movements and cramped-synchronized character of movements [27, 28, 31, 38]. An important observation from our study was the association between abnormal GMAs and PNC use. Even though our study was limited by a small sample size and small number of abnormal GMAs, this difference was significant after adjusting for gestational age, albeit only for univariable analysis. Likewise, quality of movement, repertoire, posture, and movement character differed between the PNC and no-PNC groups, suggesting that PNC may have a negative effect on early neurodevelopment. A larger multisite sample size may demonstrate a more significant effect to support this finding.

Our study reported a lower median MOS and a higher frequency of abnormal assessments in the PNC recipient group than in the no-PNC group, although this difference was not statistically significant. A small sample size may have accounted for this finding. However, this observation was consistent with the study hypothesis, and there were more infants with MOS within the severely impaired and suboptimal ranges for the PNC group than for the no-PNC group. Hitzert et al. investigated the effect of hydrocortisone versus dexamethasone on GMAs at three months post-term [25, 31]. They found that MOS scores were lower for dexamethasone, suggesting that neurological functioning was poor with dexamethasone [25, 31]. Univariable analysis from this study reports several variables, including PNC, that may contribute to abnormal GMA results; however, only the presence of PVL was associated with abnormal GMAs on multivariable analysis. Further studies are needed to explore the relationship between PNC and long-term neurodevelopmental outcomes, although it is difficult to draw causation when infants more likely to receive PNC are those who are born earlier and smaller, which already puts these infants at higher risk of poorer neurodevelopmental outcomes.

There are limited studies using Prechtl’s GMA during the fidgety period to predict neurodevelopmental outcomes following the use of PNC. There are some studies with small sample sizes that link high-dose PNC to abnormal GMAs as early as 24 hours post-administration and at three months corrected age [39, 40]. A more recent study by Hitzert et al. reported higher MOSs when low-dose and later dexamethasone was used for a shorter duration of mechanical ventilation [31]. Another study, also by Hitzert et al., reported a higher rate of abnormal GMAs for infants treated with low-dose dexamethasone and for those treated with hydrocortisone after seven days of life, with a greater rate of abnormality in the dexamethasone group [25]. It is important to note the limitations, differences in the study cohort and the methodology between this and our study. In the context of very limited previous evidence, our findings suggest an ongoing link between PNC use and abnormal neurodevelopment that warrants further exploration.

Our study provides new information on the relationship between GMAs and systemic PNC in infants dependent on invasive and noninvasive respiratory support. Blinding raters to the infant's PNC status and their perinatal course as well using a recognized process for resolution of disagreement between raters strengthens the study methodology and the generalizability of the results. We acknowledge certain limitations. This was a single-center study with a small sample size. The proportion of loss to follow-up was higher in the no-PNC group, which may also have influenced the study findings.

In conclusion, abnormal early neurodevelopment, as indicated by abnormal GMAs, was observed in infants who received PNC. In this study, the influence of other adverse perinatal conditions or complications contributing to abnormal early neurodevelopment remains unclear. Nevertheless, the effect of PNC on early neurodevelopment needs further exploration. Until further evidence is available, the use of systemic PNC is best reserved as per the current international recommendations.

## Data Availability

All data generated or analyzed during this study are included in this published article.
